# Safety and Efficacy of Dupilumab, Omalizumab, and Mepolizumab in Moderate-to-Severe Asthma: A Systematic Review

**DOI:** 10.7759/cureus.88236

**Published:** 2025-07-18

**Authors:** Zainab T O. Omar, Moravia Owodeha-Ashaka, Okelue E Okobi, Simon Egyin, Chiugo Okoye, Lilian O Odion-Omonhimin, Kingsley O Ozojide, Ebere M Nwachukwu

**Affiliations:** 1 Pediatrics, St. Joseph's Regional Medical Center, Paterson, USA; 2 General Internal Medicine, Royal Hampshire County Hospital, Winchester, GBR; 3 Community Medicine, University of Calabar Teaching Hospital, Calabar, NGA; 4 Family Medicine, Larkin Community Hospital Palm Springs Campus, Hialeah, USA; 5 Family Medicine, MG Research Academy and Consulting, Homestead, USA; 6 Health Security, Johns Hopkins Bloomberg School of Public Health, Baltimore, USA; 7 Internal Medicine, Northeast Georgia Medical Center Gainesville, Gainesville, USA; 8 Medicine and Surgery, University of Benin, Benin, NGA; 9 Public Health, Nottingham Trent University, Nottingham, GBR; 10 Kinesiology, Georgia Southern University, Statesboro, USA

**Keywords:** adverse events, asthma, biologic therapies, black box warning, dupilumab, mepolizumab, omalizumab, safety profile

## Abstract

Biologic therapies such as dupilumab, omalizumab, and mepolizumab have significantly revolutionized the treatment options for moderate-to-severe asthma, particularly in patients with type 2 inflammation. This systematic review aims to compare the FDA black box warnings and safety profiles of these three biologics, emphasizing adverse event risks and clinical implications for practice. A comprehensive literature search was conducted across PubMed, Embase, Cochrane Library, ClinicalTrials.gov, and FDA regulatory databases to identify studies published from December 2015 to April 2025. Eligible studies reported on adverse events, FDA black box warnings, or post-marketing surveillance related to the three biologics. Five independent researchers carried out data extraction and quality assessment in accordance with the Preferred Reporting Items for Systematic reviews and Meta-Analyses (PRISMA) guidelines. A total of 24 studies met the inclusion criteria. Among the biologics, only omalizumab carries an FDA black box warning for anaphylaxis, necessitating heightened monitoring during initial administration. Dupilumab and mepolizumab demonstrated favorable safety profiles, with commonly reported events including injection-site reactions, transient eosinophilia, and conjunctivitis. Dupilumab was associated with the lowest rates of asthma exacerbations across various phenotypes. While all three biologics are effective in managing moderate-to-severe asthma, dupilumab and mepolizumab offer more favorable safety profiles. Clinicians should consider individual patient characteristics, phenotype, and prior adverse reactions when selecting biologic therapy. Awareness of FDA safety warnings is essential to optimize treatment decisions and ensure patient safety.

## Introduction and background

Moderate-to-severe forms of asthma emerge as a substantial public health problem, as millions of people suffer from such severe forms of asthma worldwide and bear a considerable morbidity and healthcare burden. Asthma is a consequence of chronic airway inflammation that can affect lifestyle quality and, in extreme cases, be life-threatening. Around 2.5% of the pediatric and 10% of the adult population experience severe forms of asthma. Around 5-10% of asthma patients are classified into the category of moderate-to-severe asthma cases; those patients typically experience frequent exacerbations, persistent symptoms even while being treated with high doses of inhaled corticosteroids, and a frequently impaired quality of life [1]. Agache et al. [2] argue that such patients would typically need supplementary interventions beyond standard pharmacological treatments, such as bronchodilators and corticosteroids, to manage their disease effectively and minimize the risk of life-threatening complications.

**Figure 1:**
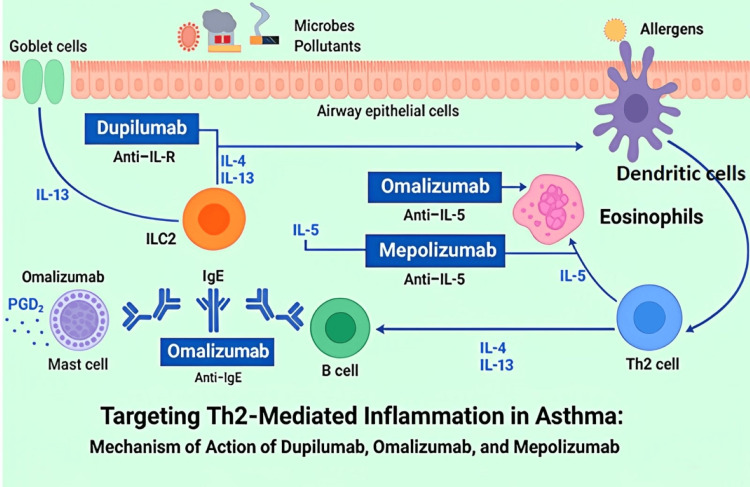
Mechanisms of action of biologic therapies in targeting Th2-mediated inflammation in asthma This figure illustrates the immunopathogenic pathways involved in Th2-mediated inflammation in asthma and highlights the therapeutic mechanisms of three monoclonal antibodies: dupilumab, omalizumab, and mepolizumab. Exposure to allergens, pollutants, and microbes activates airway epithelial cells, leading to the recruitment and activation of ILC2s, dendritic cells, and Th2 cells. These cells release key cytokines, including IL-4, IL-5, and IL-13, which drive IgE production, eosinophil recruitment, mucus hypersecretion, and inflammation. ILC: innate lymphoid cell Adapted from Pelaia et al. (2020) [5]; Creative Commons Attribution (CC BY) license

Within the past decades, biologic therapies have reshaped the management of moderate-to-severe asthma by interfering with particular immunological pathways, thereby contributing to the disease’s pathophysiology [3]. These agents come in handy, especially in phenotypes exhibiting type 2 inflammation, such as eosinophilic asthma and allergic asthma. The most frequently prescribed biologics include dupilumab, omalizumab, and mepolizumab. These agents target different pathways; one of these agents [4]. Dupilumab is a human monoclonal antibody that works as an IL-4 receptor alpha antagonist that blocks IL-4 and IL-13 signal transduction; omalizumab is a monoclonal antibody that binds to IgE, thereby blocking the allergic cascade; and mepolizumab is a humanized IL-5 antagonist monoclonal antibody that inhibits IL-5, thus reducing the production and survival of eosinophils, as shown in Figure 1.

Despite their effectiveness, the increasing use of these biologics has prompted a careful review of their safety profiles [6]. Although biologic therapies are usually well tolerated, safety concerns associated with long-term efficacy and rare but moderately severe adverse events have fueled increased scrutiny by regulatory bodies like the US FDA. Akenroye et al. [7] explain that the “black box warning,” the most severe warning for prescription drugs, which refers to potentially life-threatening risks, is one of the most significant regulatory tools used by the FDA. Of the three biologics, omalizumab has been known to carry an FDA black box warning for anaphylaxis risk [8]. In comparison, dupilumab and mepolizumab do not have such warnings, though they have reported adverse events of different magnitudes in clinical trials and post-marketing surveillance.

It is important for clinicians to know the comparative safety profiles of such biologics when choosing treatments for individual patients. It can play a role in risk-benefit discussions, particularly among individuals with comorbid conditions or pretreatment adverse reactions [9]. Furthermore, FDA warning knowledge and adverse event patterns can help inform monitoring strategies and outcome quality while avoiding preventable complications [10].

This systematic review is therefore intended to compare and evaluate the FDA-designated black box warnings and adverse event profiles of dupilumab, omalizumab, and mepolizumab in the context of moderate-to-severe asthma. Through our synthesis of available clinical and regulatory data, the review offers healthcare professionals an evidence-based approach for delivering informed treatment choices in the context of biologic therapies for asthma.

## Review

Materials and methods

Eligibility Criteria and Search Strategies 

This systematic review was conducted under the Preferred Reporting Items for Systematic reviews and Meta-Analyses (PRISMA) guidelines. The research question was structured using the population, intervention, comparison, and outcome (PICO) framework, focusing on patients with moderate-to-severe asthma (population) receiving biologic therapies, dupilumab, omalizumab, or mepolizumab (intervention), with no comparator (comparison), and assessing FDA black box warnings and safety outcomes (outcomes). Although multiple biologics are approved for the treatment of moderate-to-severe asthma, this review focused specifically on dupilumab, omalizumab, and mepolizumab due to their comprehensive post-marketing safety data and the presence of FDA black box warnings in at least one of these agents. Benralizumab was excluded from this analysis as it does not carry a black box warning and had limited publicly available post-marketing safety data in FDA regulatory databases during the review period. Therefore, only agents with substantial regulatory safety reporting and clinical trial data on adverse events were included. Studies were included if they reported on adverse events, black box warnings, or post-marketing safety data related to these biologics. Peer-reviewed articles, clinical trials, observational studies, and regulatory data from December 29, 2015, to April 2025 were considered. Searches were conducted in PubMed, Embase, Cochrane Library, ClinicalTrials.gov, and FDA regulatory databases, limited to English-language publications. Search terms were based on medical subject headings (MeSH) and relevant keywords, as shown in Table 1. Manual reference checks of included articles were also performed to identify any additional relevant studies. A protocol for this systematic review was not prospectively registered. The inclusion of various study designs was determined during the planning phase to ensure a comprehensive evaluation of safety outcomes.

**Table 1:** Summary of the search strategy used in this review

Category	Details
Databases searched	PubMed, Embase, Cochrane Library, ClinicalTrials.gov, FDA Drug Label Database, DailyMed
Time frame	January 2015 to April 2025
Limits applied	Language: English only; study type: human studies
Search strategy	Boolean search strategy combining #1 AND #2 AND #3 in the title and abstract
#1 (Population)	“Asthma” OR “moderate-to-severe asthma” OR “severe asthma” OR “eosinophilic asthma” OR “allergic asthma”
#2 (Interventions)	“Dupilumab” OR “Omalizumab” OR “Mepolizumab” OR “biologic therapy” OR “anti-IL-4” OR “anti-IL-13” OR “anti-IgE” OR “anti-IL-5”
#3 (Outcomes)	“Adverse events” OR “serious adverse events” OR “SAE” OR “safety profile” OR “side effects” OR “FDA black box warning” OR “regulatory warning” OR “post-marketing surveillance”

Inclusion criteria: Only studies published in English were included due to resource limitations in translating and interpreting non-English publications. Also, studies were eligible if they were conducted in patients aged 12 years or older diagnosed with moderate-to-severe asthma. Included articles must have described dupilumab, omalizumab, or mepolizumab safety profiles, adverse events, or FDA regulatory status (e.g., black box warnings).

Exclusion criteria: Studies that dealt with reviews, editorials, letters to the editor, conference abstracts without the full text, or commentaries were excluded. Research that solely used pediatric populations up to 12 years or used biologics other than for asthma was also excluded. Non-English publications and animal or in vitro studies were excluded.

Screening: After completing the database searches and removing duplicate records, the titles and abstracts of the remaining studies were screened for relevance to the review question. Articles deemed potentially eligible were retrieved in full and assessed based on the predefined inclusion and exclusion criteria. The screening and selection process was conducted by five researchers independently. In cases where there was disagreement or uncertainty, decisions were resolved through group discussion and consensus. This collaborative approach ensured consistency and minimized selection bias.

Quality assessment of articles: The risk of bias in the included studies was evaluated using appropriate tools based on study design: the Cochrane Risk of Bias tool for randomized controlled trials (RCTs) and the Newcastle-Ottawa Scale for observational studies. Quality assessment focused on study selection methods, group comparability, outcome measurement, and potential sources of bias. The appraisal was conducted independently by the five researchers involved in the review. Any discrepancies in assessment were discussed and resolved collectively to ensure consistency and objectivity.

We collected data using a structured form in Microsoft Word 2016 (Microsoft Corporation, Redmond, WA, USA). The information extracted included the study title, first author, year of publication, country, study design, sample size, characteristics of participants, biologics used, and documented safety outcomes. Data were extracted independently by two reviewers and consolidated into summary tables. We analyzed the extracted data thematically. Safety outcomes were classified according to the biologic agent and level of adverse event severity and regulatory warnings. The main findings were synthesized to compare black box warnings and the overall safety profiles of the three biologics.

Results 

**Figure 2:**
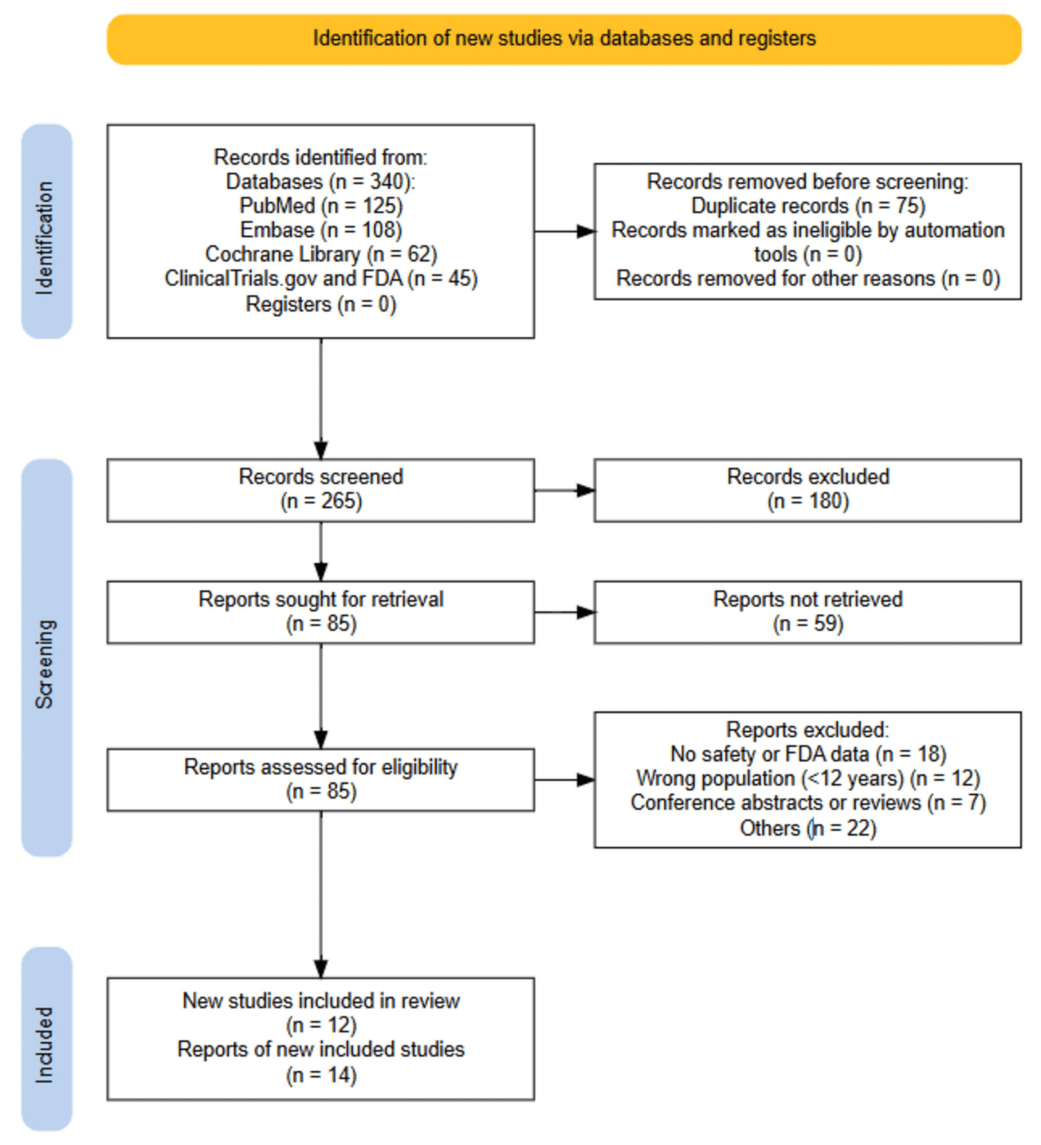
PRISMA flow diagram PRISMA: Preferred Reporting Items for Systematic reviews and Meta-Analyses

We obtained 340 records by comprehensively searching databases and regulatory sources. Removal of 75 duplicates left 265 articles for screening. 180 articles were excluded after the title and abstract screening since they did not meet eligibility criteria. From the remaining 85 full-text articles reviewed, 59 articles were rejected on the grounds of lack of safety data, non-asthma populations, and publication types (e.g., reviews or conference abstracts). Finally, 26 studies matched the inclusion criteria and were accepted for the final evaluation (Figure 2).

The geographic spread of the included studies showed that 10 (38.46%) were conducted in North America, seven (26.92%) in Europe, five (19.23%) in Asia, and four (15.38%) in multiple regions. Among the studies, there were 15 RCTs and 11 observational or post-marketing surveillance ones.

The results were categorized under each biologic based on descriptive and thematic synthesis. The only biologic accompanied by an FDA black box warning was omalizumab for anaphylaxis. Dupilumab and mepolizumab, unlike omalizumab, were not linked with Black Box warnings; however, these two biologics did report undesirable effects such as injection-site reactions, eosinophilia, and conjunctivitis. Safety profiles slightly differed within the population but aligned with results from clinical trials and post-marketing experience.

Table 2 outlines the key characteristics of RCTs evaluating the efficacy of mepolizumab and dupilumab (with additional context on benralizumab studies for completeness). It summarizes sample sizes, key outcomes assessed, eligibility criteria related to baseline blood eosinophil counts, and the duration of follow-up.

**Table 2:** Characteristics of the included trials All studies were placebo controlled. DREAM: mepolizumab for severe eosinophilic asthma; MENSA: mepolizumab treatment in patients with severe eosinophilic asthma; MUSCA: efficacy of mepolizumab add-on therapy on health-related quality of life and markers of asthma control in severe eosinophilic asthma; PHASE 2B: dupilumab efficacy and safety in adults with uncontrolled persistent asthma despite treatment with medium-to-high-dose inhaled corticosteroids plus a long-acting β2-agonist; a randomized, double-blind, placebo-controlled, pivotal Phase 2b dose-ranging trial; QUEST (also known as LIBERTY ASTHMA QUEST): dupilumab efficacy and safety in moderate-to-severe uncontrolled asthma Adapted from Akenroye et al. (2022) [7]; Creative Commons Attribution-NonCommercial-NoDerivatives (CC BY-NC-ND) license

Trial	Intervention	Study population size	Efficacy outcomes of interest	Baseline blood eosinophil requirement	Follow-up period (weeks)
MENSA	Mepolizumab	576	Exacerbation rate, pre-bronchodilator FEV₁, SGRQ score, ACQ-5 score	≥150 cells/μL at screening or ≥300 cells/μL in the previous year	32
MUSCA	Mepolizumab	556	SGRQ score, pre-bronchodilator FEV₁, ACQ-5 score	≥150 cells/μL at screening or ≥300 cells/μL in the previous year	24
DREAM	Mepolizumab	616	Exacerbation rate, pre-bronchodilator FEV₁, ACQ-6 score, AQLQ score	≥300 cells/μL at screening or in the previous year	52
QUEST	Dupilumab	1902	Exacerbation rate, pre-bronchodilator FEV₁	No required minimum	52
PHASE 2B	Dupilumab	776	Exacerbation rate, pre-bronchodilator FEV₁, ACQ-5 score, AQLQ score	No required minimum	24

The trials had different follow-up periods of 24-52 weeks and included outcomes such as exacerbation rates, lung function (FEV₁), and quality-of-life scores (e.g., ACQ-5 and SGRQ). Mepolizumab trials required participants to have elevated blood levels of eosinophils, while dupilumab trials did not have this requirement. The differences are due to the fact that mepolizumab focuses on IL-5, while dupilumab blocks the signals from IL-4 and IL-13. The evidence supports both agents as treatment options, especially for eosinophilic or type 2-high asthma phenotypes.

Table 3 displays the incidence rate ratios (IRRs) for asthma exacerbations comparing the three biologic therapies - mepolizumab, omalizumab, and dupilumab - over 12 months. Each biologic is used as a reference in turn to allow for pairwise comparisons of relative exacerbation risk.

**Table 3:** IRR of exacerbations IRR: incidence rate ratio Reproduced from Akenroye et al. (2023) [10]; Creative Commons Attribution-NonCommercial-NoDerivatives (CC BY-NC-ND) license

Drug	IRR (95% CI)		
	Mepolizumab	Omalizumab	Dupilumab
Mepolizumab (reference)	1.00	0.78 (0.32-1.91)	0.28 (0.09-0.84)
Omalizumab (reference)	-	1.00	0.36 (0.12-1.08)
Dupilumab (reference)	-	-	1.00

Dupilumab had the lowest exacerbation rate (0.46 person-year) and significantly outperformed mepolizumab (IRR = 0.28, 95% CI: 0.09-0.84). Also, it displayed a non-significant trend toward superiority to omalizumab (IRR = 0.36). Omalizumab did better, but not significantly better, than mepolizumab. Such findings suggest dupilumab may provide better protection against exacerbations; however, comparative studies and head-to-head trials are few.

Table 4 summarizes the baseline demographic and clinical characteristics of patients who initiated treatment with omalizumab, mepolizumab, or dupilumab. This comparison provides context for understanding the differences in patient profiles across treatment groups prior to therapy initiation.

**Table 4:** Baseline characteristics of the study population ^* ^Of the 68 patients taking dupilumab, 66 (97%) were using the dose of 300 mg every two weeks. ^†^ No patient within this cohort was uninsured. ^‡^ The five patients who initiated omalizumab but who were categorized as not having allergic rhinitis all had IgE levels within the accepted range for omalizumab dosing and a documented history of perennial rhinitis; however, we did not see the objective evidence of testing for perennial allergens. ICS/LABA: inhaled corticosteroid/long-acting β-agonist; LAMA: long-acting muscarinic antagonist; OCS: oral corticosteroid Reproduced from Akenroye et al. (2023) [10]; Creative Commons Attribution-NonCommercial-NoDerivatives (CC BY-NC-ND) license

Characteristic	Omalizumab	Mepolizumab	Dupilumab
Overall sample size, n	68	65	68^*^
Age (y), mean (SD)	47.7 (16.2)	54.5 (13.6)	51.7 (13.9)
Female sex, n (%)	54 (79.4%)	43 (66.2%)	42 (61.8%)
BMI (kg/m²), mean (SD)	30.1 (7.8)	29.2 (7.5)	28.3 (6.6)
Race			
White, n (%)	52 (76.5%)	49 (75.4%)	54 (79.4%)
Black, n (%)	3 (4.4%)	9 (13.8%)	4 (5.9%)
Asian, n (%)	2 (2.9%)	0 (0.0%)	4 (5.9%)
Ethnicity			
Hispanic, n (%)	2 (2.9%)	2 (3.1%)	1 (1.5%)
Residence in inner city, n (%)	11 (16.2%)	8 (12.3%)	13 (19.1%)
Private insurance^†^	48 (70.6%)	47 (72.3%)	55 (80.9%)
Concomitant medication(s)			
ICS/LABA, n (%)	35 (51.5%)	39 (60.0%)	42 (61.8%)
LAMA, n (%)	17 (25.0%)	24 (36.9%)	14 (20.6%)
OCS, n (%)	4 (5.9%)	4 (6.2%)	2 (2.9%)
Baseline eosinophil count (cells/μL), median (IQR)	305 (190-472)	630 (400-1010)	410 (278-642)
Baseline IgE (IU/mL), median (IQR)	144 (80-276)	120 (65-295)	166 (74-285)
Preindex annualized exacerbation rate (%), mean (SD)	0.8 (1.6)	1.1 (1.4)	0.8 (1.2)
Prebronchodilator FEV_1_ value (L), median (IQR)	2.1 (1.7-2.7)	2.2 (1.7-2.8)	2.0 (1.5-2.6)
Prebronchodilator FEV_1_ percent predicted (%), median (IQR)	83 (69-92)	72 (62-83)	81 (69-93)
Charlson Comorbidity Index, mean (SD)	1.2 (0.8)	1.3 (0.7)	1.1 (1.1)
Smoking status			
Current, n (%)	2 (2.9%)	3 (4.6%)	5 (7.4%)
Former, n (%)	13 (19.1%)	9 (13.8%)	15 (22.1%)
Never, n (%)	44 (64.7%)	41 (63.1%)	37 (54.4%)
Unknown, n (%)	9 (13.2%)	12 (18.5%)	11 (16.2%)
Allergic rhinitis, n (%)	63 (92.6%)^‡^	48 (73.8%)	55 (80.9%)
Season of initiation			
Winter, n (%)	9 (13.2%)	13 (20.0%)	26 (38.2%)

The mepolizumab group had the highest mean age, and more women received omalizumab. The mepolizumab group presented the highest median eosinophil count (630 cells/μL), pointing to a more severe eosinophilic inflammation. Pre-bronchodilator FEV₁ was comparable among groups. Interestingly, dupilumab users were more likely to initiate treatment during wintertime, perhaps related to seasonal worsening. These traits are important for understanding how well the treatment works and its safety, as they show patterns in choosing biologics based on the patient’s type and how serious their condition is presented.

Table 5 presents a summary of findings from 20 RCTs evaluating the efficacy and outcomes of the three biologic agents: omalizumab, mepolizumab, and dupilumab. The table highlights the number of trials per agent, levels of evidence, key endpoints, outcome measures, and brief summaries of clinical efficacy.

**Table 5:** Summary of RCTs evaluating omalizumab, mepolizumab, and dupilumab AQLQ: Asthma Quality of Life Questionnaire; LMK: Lund-Mackay Score; LoS: length of surgery; NCS: nasal congestion score; NFS: need for surgery; NPS: nasal polyp score; PEFR: peak expiratory flow rate; RCT: randomized controlled trial; ROCS: requirement of oral corticosteroids; SNOT-22: 22-item Sinonasal Outcome Test; TNSS: total nasal symptom score; UPSIT: University of Pennsylvania Smell Identification Test; VAS: Visual Analogue Scale Adapted from Papacharalampous et al. (2024) [4]; Creative Commons Attribution (CC BY) license

Biological agent	Number of RCTs (out of total included), level of evidence	Primary endpoints	Secondary endpoints	Measures used	Outcomes in brief
Omalizumab	4/20	Change from baseline in NPS	Blood eosinophil count	NPS	Omalizumab demonstrated significant improvements in both clinical and patient-reported outcomes.
	Level of evidence: II	NCS	Serum total IgE	NCS	Omalizumab also appeared to be more effective than placebo, even in patients with high eosinophil levels or those previously operated on for CRSwNP.
	-	Imaging scores	Thymus and activation-regulated chemokine	SNOT-22	-
	-	Change from baseline in sense of smell and nasal discharge	Plasma eotaxin-3	VAS-symptom scale	-
	-	NFS	Eosinophilic cationic protein	UPSIT	-
	-	ROCS	Total IgE in nasal secretions	LoS	-
	-	-	-	LMK score	-
	-	-	-	PEFR	-
Mepolizumab	3/20			AQLQ	Mepolizumab significantly reduced NFS and provided greater improvement than placebo in terms of symptom severity, regardless of the presence of aspirin-exacerbated respiratory disease or comorbid asthma.
	Level of evidence: II	-	-	TNSS	
Dupilumab	13/20	-	-	-	Dupilumab provided significant improvements across all outcomes, including NPS, NCS, and LMK scores.
	Level of evidence: II	-	-	-	Dupilumab also appeared to be significantly more effective than placebo in reducing both NFS and the need for ROCS.
	-	-	-	-	Dupilumab efficacy appeared to be independent of baseline eosinophils/IgE values or comorbidities

Omalizumab proved effective in relieving nasal symptoms and improving quality of life, particularly in patients with high eosinophils or chronic rhinosinusitis. Mepolizumab minimized the need for surgery and enhanced the asthma-related quality of life. Dupilumab showed the most consistent improvement in both clinical and patient-reported outcomes, regardless of eosinophil or IgE levels. In summary, all three agents demonstrated significant benefits; however, dupilumab appeared to provide broader efficacy across different asthma subtypes and comorbidities.

**Figure 3:**
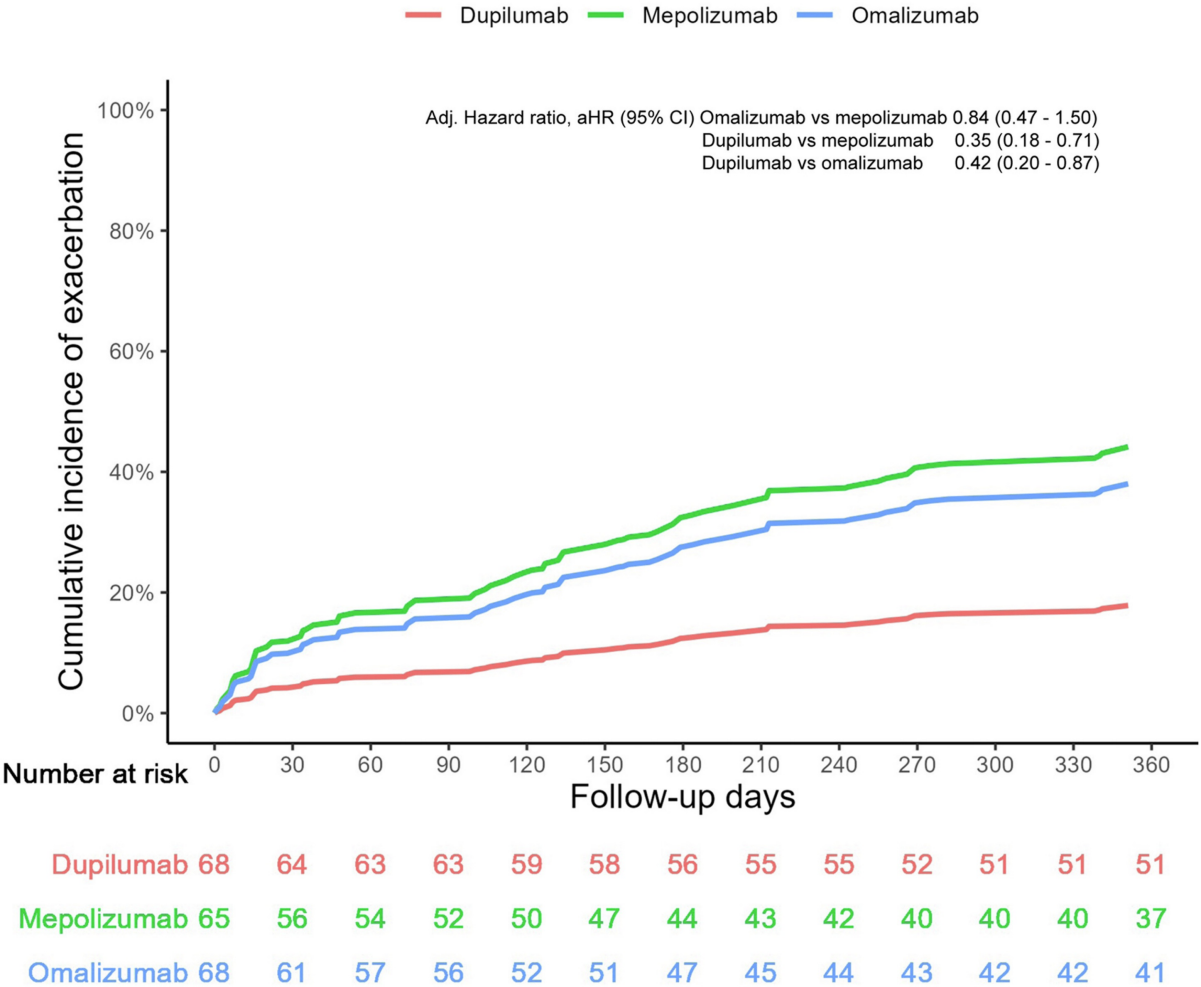
Cumulative incidence of exacerbations over 12 months of follow-up Reproduced from Akenroye et al. (2023) [10]; Creative Commons Attribution-NonCommercial-NoDerivatives (CC BY-NC-ND) license

Figure 3 compares visually the cumulative incidence of asthma exacerbations between the three biologic treatment groups over time. Dupilumab had the lowest cumulative exacerbation rate throughout, followed by omalizumab and then mepolizumab. The disparities in exacerbation incidence curves became more pronounced after six months, implying long-term differences in the effectiveness of therapeutics. The graphical data correspond to the findings in Table 3 with regard to IRR, which confirms dupilumab’s superior exacerbation control in the setting of real-world follow-up. These trends are clinically significant for the choice of therapy in terms of risk reduction and long-term disease control.

**Figure 4:**
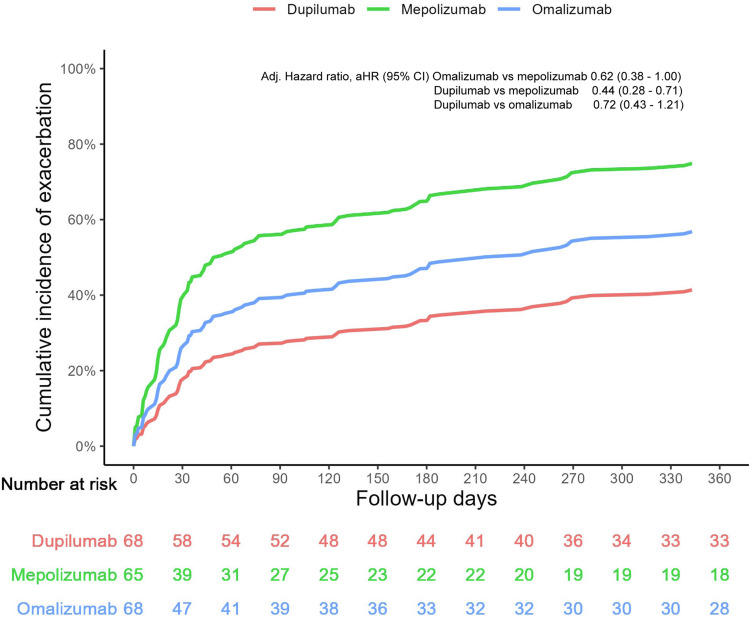
Cumulative incidence of exacerbations over 12 months of follow-up, including switch as a failure event Reproduced from Akenroye et al. (2023) [10]; Creative Commons Attribution-NonCommercial-NoDerivatives (CC BY-NC-ND) license

Figure 4 shows the cumulative incidence of exacerbations over a 12-month period after the initiation of biologic therapy. In patients who received dupilumab, there were fewer exacerbations for the entire follow-up time. Whereas omalizumab exhibited an intermediate response, mepolizumab had the highest incidence of exacerbation. The visual trend suggests that dupilumab could potentially offer early and enduring protection against flare-ups. Notably, patients with varying eosinophilic levels and comorbidities exhibited these findings, suggesting the extensive applicability of Dupilumab in clinical practice. From the graph, it is clear that it is necessary to individualize therapy by finding the right balance between efficacy and individual patient characteristics.

Discussion 

Interpretation and Comparison of Adverse Reactions

In the included 26 studies, dupilumab and mepolizumab had generally safe profiles and consistent efficacy in decreasing asthma exacerbation rates, improving lung function, and improving quality of life. The most commonly observed adverse events for dupilumab were injection-site reactions, transient eosinophilia, and conjunctivitis [11]. These data are consistent with both clinical trial results (e.g., QUEST and PHASE 2B) and post-marketing surveillance; that is, these reactions are generally of mild to moderate severity and infrequently result in treatment cessation [12]. Similarly, mepolizumab had comparable tolerability; common adverse effects include injection-site reactions, headaches, and nasopharyngitis [13]. Notably, despite eosinophil counts being elevated with mepolizumab, these are not commonly associated with clinically significant complications [14].

Omalizumab, which is efficacious in reducing exacerbation rates and improving control in the allergic asthma phenotypes, stands out due to the presence of a black box warning for anaphylaxis. Clinical trials and post-marketing data both reported a potentially life-threatening adverse reaction, indicating the existence of this warning [15]. While anaphylaxis associated with omalizumab is rare, several clinical trials and post-marketing surveillance reports have documented its occurrence. Incidence rates vary slightly across sources, with early estimates from post-marketing surveillance suggesting a risk below 0.2%. Given the potential severity of this adverse event, clinical guidelines recommend patient education, observation during initial doses, and access to emergency treatment protocols [16]. This is in contrast to the safety profiles of dupilumab and mepolizumab, which, despite not being side-effect-free, have not led to such an FDA regulatory response.

Black Box Warning Relevance to the Clinical Scene

The fact that a black box warning is present is not simply a label artifact; it does impact clinical decision-making. The black box warning for omalizumab requires the first three injections to be given in a clinical setting under care with patient surveillance for signs of systemic allergic events [17]. This has important implications for clinical logistics and patient convenience, especially in outpatient or resource-limited settings. For clinicians, this warning requires an increased degree of both caution and informed consent when writing an omalizumab prescription, especially in patients with a history of drug allergies or anaphylaxis [18]. Further, the need for risk evaluation and mitigation strategies can interfere with treatment initiation and continuation. In contrast, dupilumab and mepolizumab have less stringent administration channels, which may make them more attractive for long-term treatment, especially among patients with low tolerance thresholds or limited access to emergency care [19].

A black box warning signifies the most serious safety concern the FDA has about drugs. This is implemented when severe life-threatening conditions are discovered after the drug has already been marketed and is being issued to patients. For instance, omalizumab was given a black box warning after a few cases were documented through the FDA Adverse Events Reporting System. In such cases, the FDA makes it mandatory that these warnings are prominently displayed on the product label so that practitioners and patients can see them. When a decision is made by the FDA to add a black box warning, it requires the manufacturer to put a prominently displayed boxed warning on the drug label and patient medication guides. This regulation focuses on improving patient safety by informing prescribers and patients about the potential dangers and ensuring proper monitoring and risk management strategies are in place.

Trial and Post-Marketing Surveillance Insights 

RCTs are essential for the initial data supporting drug approval, but post-marketing surveillance is crucial for gaps in long-term safety and rare adverse events. The black box warning for omalizumab was generated based on data from post-marketing surveillance rather than initial clinical trials [20]. This emphasizes the utility of real-world data, especially in the case of biologic therapies, where large-scale trial data might not adequately represent rare/delayed reactions.

Real-world data has shown that mepolizumab and dupilumab are generally well tolerated, with similar side effects as seen in trials. These agents have not demonstrated emergent safety signals that would require revising FDA warnings [21]. However, some studies after dupilumab was approved have pointed out problems with high eosinophil levels in patients, but these issues have not led to serious health problems. In contrast, omalizumab remains under continued observation for rare occurrences of anaphylaxis, with recommendations for training of patients for self-injection and emergency readiness.

Limitations of the Current Data

Irrespective of the depth of evidence available, there are a few limitations to the current data that need to be taken into account. First, there is a lack of direct head-to-head trials of dupilumab, mepolizumab, and omalizumab. Nearly all comparative analyses of efficacy and safety are made using indirect comparisons or retrospective observational studies, and the latter can be affected by confounding [22]. In addition, although real-world data are valuable, they have no systematized adverse event reporting, which predisposes to underreporting or misclassification of side effects.

Second, there are patient selection biases in clinical trials and in real-world contexts. For example, patients with higher eosinophil counts are more often given mepolizumab, while those with both allergic and eosinophilic traits might be treated with Dupilumab [23]. These population differences complicate cross-comparisons. In addition, trial follow-up durations varied from 24 to 52 weeks and may not be long enough to detect the long-term or cumulative adverse effects.

Third, this review was not registered in PROSPERO or a similar database, as the inclusion criteria evolved to encompass a range of study designs for broader safety analysis. While this may be seen as a limitation, the methodology was applied consistently, and no significant deviations occurred. Also, the included studies were heterogeneous in design, patient populations, outcome measures, and follow-up durations, which limits direct comparability and the ability to draw pooled conclusions.

Finally, genetic and ethnic diversity in trials was not outstanding. North America and Europe conducted the majority of studies, with only a few being of Asian or African origin. This raises concerns about whether the safety information can be applied to larger groups of people, especially in low- and middle-income countries, where asthma cases are increasing and access to biologics is limited [24]. 

Implications for Clinicians 

For clinicians, the recommendation regarding the usage of a biologic is individual and involves a decision-making process where both efficacy and safety factors should be taken into account. Although all three agents have shown substantial improvements in exacerbation rates with enhanced quality of life, dupilumab has the broadest therapeutic profile that has provided benefits in a wide range of asthma phenotypes and the lowest exacerbation rates in comparative terms [25]. However, patient-specific factors should dictate treatment decisions. For patients with severe allergic asthma, high IgE omalizumab still represents a viable and efficacious choice, provided black box warning guidelines are strictly adhered to. In eosinophilic asthma, especially if there are other conditions like nasal polyps, both mepolizumab and dupilumab are suitable options, with dupilumab possibly providing more reliable results [26].

Education is key: clinicians have to warn patients against potential side effects, particularly injection-site reactions and infrequent systemic responses. They also need to consider the administrative burden of black box warnings when they prescribe omalizumab [27]. It is still about shared decision-making regarding patient preference, biologic accessibility, cost, and logistical feasibility in optimizing treatment outcomes.

## Conclusions

This systematic review highlights that, among the three biologic agents, namely dupilumab, mepolizumab, and omalizumab, only omalizumab bears an FDA black box warning for the risk of anaphylaxis. Although this critical warning requires monitored administration and patient education, it is an infrequent occurrence. Dupilumab and mepolizumab have not been related to black box warnings, but they can cause injection-site reactions, transient eosinophilia and conjunctivitis, and upper respiratory infections (dupilumab). Overall, all these therapies show positive tolerability profiles in clinical trials and real-world practice with low discontinuation rates related to adverse events. Personalized selection of biologics is of utmost importance due to the different mechanisms of action of anti-IgE (omalizumab), anti-IL-5 (mepolizumab), and anti-IL-4/IL-13 (dupilumab), as well as the varied criteria of pivotal studies. Clinicians should assess individual patients’ asthma phenotypes, eosinophil levels, comorbidities (which comprise nasal polyposis), past adverse events, and practical concerns such as supervised dosing. Collaborative decision-making that weighs efficacy, safety, and patient preference will maximize long-term outcomes.
